# The role of Vesalius and his contemporaries in the transfiguration of human anatomical science

**DOI:** 10.1111/joa.13773

**Published:** 2022-09-29

**Authors:** Jinpo Xiang, Santhana Venkatesan

**Affiliations:** ^1^ Human Anatomy Unit, Faculty of Medicine Imperial College London London UK; ^2^ School of Medicine Keele University Newcastle under Lyme UK

**Keywords:** anatomy, anatomy teaching, Andreas Vesalius, Berengario di carpi, education, Mondino di Luzzi, renaissance anatomy

## Abstract

The understanding of human anatomy has been an endeavour spanning thousands of years from the Egyptians and Greeks in antiquity to the present day. Scholars and scientists have overcome great barriers to discover the inner workings and complexities of the human body, from personal challenges and prejudices to obstacles placed by society. Our present understanding of anatomy has accumulated over centuries, and progressive generations of physicians have contributed to the ever‐growing evidence‐based knowledge. This article explores the contributions made by Vesalius and his contemporaries in the first half of the sixteenth century. These enlightened scholars advanced anatomical knowledge and, perhaps more importantly, the scientific method, directly impacting the mindset and methodologies of future anatomists. Individuals such as Berengario da Carpi and Gabriele Falloppio produced bodies of work during their lifetime that were not only important in disputing the teachings of Galen of Pergamon, which had been accepted as almost unquestionable truths for a thousand years, but also instrumental in developing a new generation of scientists. The anatomists of the late renaissance were unable to resolve many of the factual inaccuracies of Galenic teaching but provided the groundwork for scientific thinking which future generations of anatomists benefited greatly from. The principles of documenting what is observed and establishing a methodical approach to question discrepancies in experiments would go on to influence physicians such as Harvey and Malpighi to investigate and draw correct conclusions in their research and ultimately advance our understanding of human anatomy to what it is today.

## Graphical abstract

Andreas Vesalius as illustrated by Jan van Kalkar.

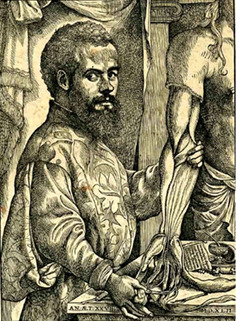

## 1 INTRODUCTION

The complexities and inner workings of the human body have been explored across millennia. The Greeks and Romans had a steady interest in the functionalities of the human body. Before the third century BCE, there is little evidence to suggest human dissection took place (Von Staden, 1992). These acts were deemed to be desecration and violated the accepted cultural boundaries (Lloyd, 1973).

**FIGURE 1:**
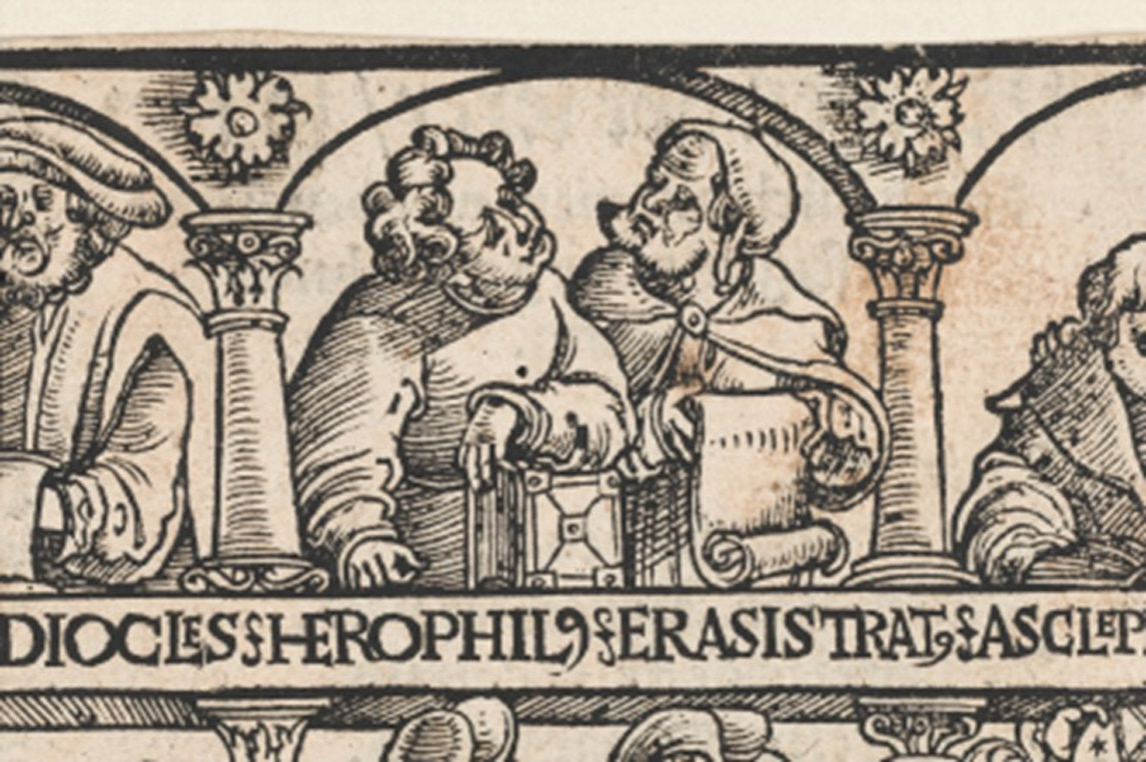
Science museum (2022). Herophilus and Erasistratus. [image] Available at: http://broughttolife.sciencemuseum.org.uk/broughttolife/people/herophilusofchalcedon [accessed 6 august 2022].

Greek anatomist Erasistratus was famous for distinguishing the differences between arteries and veins, despite claiming arteries only contained air (Aird, 2011). Comparably, Herophilus was famous as the first documented individual to note the difference between the cerebrum and the cerebellum. He investigated the cranial nerves, describing the optic nerve and oculomotor nerve for eye movement (Wills, 1999) and taught that the ‘seat of intelligence was in the brain not the heart’ (Hunter, 1931) contrary to the teachings of Aristotle (Figure 1).

The work of Galen of Pergamon formed the bulk of western knowledge on the structures and functions of the human body for over a thousand years. Galen's study of human anatomy was in bulk based on animal dissection (Ajita, 2015) as well as observations from his role as the chief physician to the gladiators in Pergamum for 4 years (Robinson, 2013). Much of the basis of anatomical teaching stemmed from the writings of Galen and records of his experimentations with animals (Derenne et al., 1995). As societal shifts focussed more on scientific observation and scholarship in the latter parts of the Middle Ages, anatomists began to regularly perform dissection for the purpose of medical education.

One of the key figures of renaissance anatomy was Andreas Vesalius. Born in Brussels in 1514 to a family with a strong medical background and ties to the Holy Roman Emperors' court (Dunn, 2003). He began medical training at the University of Louvain, obtaining his medical degree at the University of Padua in 1537. From there, he lectured in surgery and began forming the foundations of a lifetime dedicated to the study and teaching of human anatomy. Although contemporaries and predecessors to Vesalius had published works relating to anatomical teaching, Vesalius' work ‘De Humani Corporis Fabrica’ has become widely recognised as a landmark point in the methodology and teaching of anatomical science.

This article explores how the Renaissance anatomists, in particular Andreas Vesalius, impacted the study of the human body. We will address current gaps in the literature to piece together how the work of Vesalius and his contemporaries, such as Berengario di Carpi and Fallopio integrated with the ongoing transformation of European universities and their medical teaching during this period as well as the interventions and ever‐changing influence of society and the church. The article aims to understand how Vesalius held a leading role in changing teaching, what part church and society played in the evolving field of scientific anatomy and how the early universities harboured the practice of scientific observation which ultimately led towards evidence‐based medicine.

## 2 METHODS

Evidence and points of view have been collated across numerous sources including books and journal articles from both scientific and humanities research has been utilised to obtain an accurate and in‐depth understanding of the subject matter so that analytical commentary can be formed with grounding. Where possible, original material has been used, including digitally scanned sections of Vesalius' original publications and Carpi's ‘Isagogae Breves’.

## 3 DISCUSSION

### 3.1 Anatomy in the early renaissance

In the early fourteenth century, the first widely accessed book on human anatomy was created by Mondino di Luzzi (1276–1326) who was a professor of anatomy at the University of Bologna. Mondino was the first documented person since the time of Galen to have dissected a human body. This dissection took place in 1315 on an executed female criminal and was attended by medical students and the public. The purpose was to define the positioning of the anatomical elements as described by Galen (Mavrodi & Paraskevas, 2014). Dissection would consist of a professor seated above the dissecting table, reading out from the works of Galen as demonstrators, often barber surgeons, proceeded with the practical aspects of dissection. When cadaveric demonstrations did not match Galen's description, it would be proclaimed that the cadaver was abnormal or had ‘morphological transmutations’ (Frati et al., 2006).

By 1316, Mondino di Luzzi published a textbook of anatomy, ‘De omnibus humani corporis interioribus membris anathomia’. The publication was a manual for dissection, methodically processing the body starting with the abdominal cavity and finishing with the upper and lower limbs. Multiple original observations are noted, Mondino describes the cervix of the uterus in detail and noted the hymen ‘In uirginibus est uellatum uellamine subtili uenoso et antur rumpitur et ideo sanguinantur’ translated as ‘in virgins is covered by an intact subtle and venous covering, when broken, women bleed on that account’ (Kelly, 2014).

**FIGURE 2:**
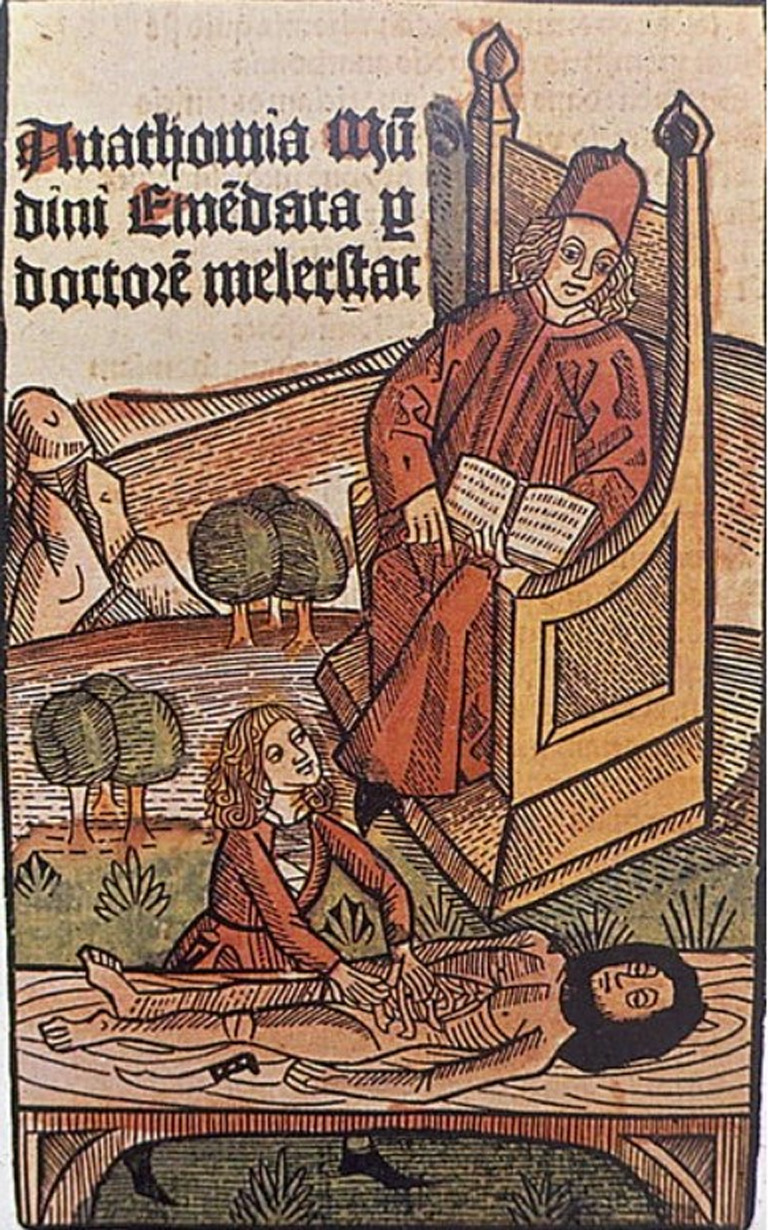
Di Matteo, B., Tarabella, V., Filardo, G., Mosca, M., Lo Presti, M., Viganò, a., Tomba, P. and Marcacci, M. (2017). Art in science: Mondino de’ Liuzzi: The restorer of anatomy. *Clinical Orthopaedics and related research®*, 475(7), pp.1791–1795.

**FIGURE 3:**
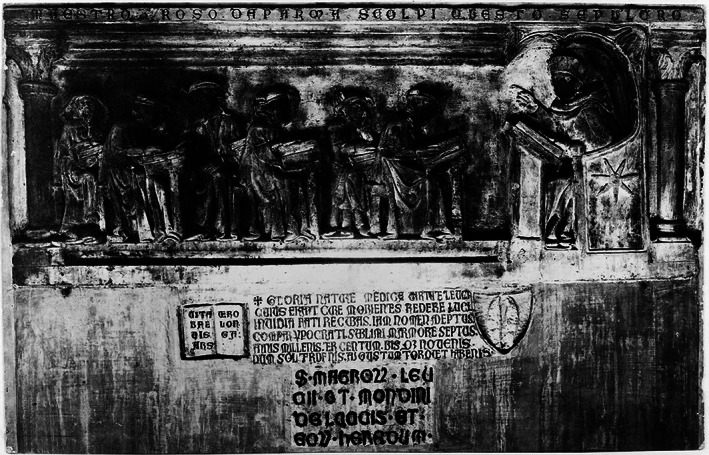
Roso da Parma, *Wellcome collection catalogue*, https://wellcomecollection.org/works/puf9kdgs, [accessed 7 august 2022].

Although Mondino's ‘Anathomia’ suggests the professor had firsthand experience of dissection, this would have not likely been the norm given his senior academic position. Mondino di Luzzi should be credited for helping establish Bologna as a prominent centre for anatomy during the Middle ages. He repeated many of Galen's incorrect theories, through unquestioned acceptance of Galen's work and his own misinterpretations in a quest to align what he observed with the teachings of Galen (Mavrodi & Paraskevas, 2014). However, his incorporation of dissection as part of medical training, and the publication of the ‘Anathomia’ enabled an environment for advances in anatomical understanding for his successors in Bologna (Figures 2 and 3).

### 3.2 Anatomy incorporated into the medical curriculum

Despite the renewed interest in the anatomical study, the teaching method and content had not changed since the time of Mondino di Luzzi. Andreas Vesalius in his preface to ‘De Humani Corporis Fabrica’ described this learning process: ‘A detestable ceremony in which certain persons are accustomed to perform a dissection of the human body while others narrate the history of the parts: these latter from a lofty pulpit and with egregious arrogance sing like magpies of things they have no experience, but rather commit to memory from the books of others; the former, so unskilled in languages, that they are unable to describe to the spectators what they have dissected’ (Vesalius, 1543).

Whilst teaching methods continued to be stagnant, the importance of anatomy to form part of the medical curriculum grew. In the large Italian cities with established universities such as Bologna, Padua and Florence, dissection became a normalised part of the medical curriculum and in fact, during the mid‐fourteenth century, these universities enforced compulsory attendance to dissections to receive a doctorate degree in medicine (Ghosh, 2015). With this growing focus on anatomy as an integrated part of medicine and surgery, a more scrutinising and observational methodology was adopted.

### 3.3 Questioning the status quo

Physicians began comparing their observed anatomy with the teachings of antiquity and began to question the discrepancies. Berengario da Carpi (1470–1530), a physician who was held in high esteem during his lifetime, published a book ‘Anatomia Carpi. Isagoge breves perlucide ac uberime, in Anatomiam humani corporis’. This book was the first to be printed with both text and illustrations to aid the reader. However more importantly, for the first time we see in print, challenges to accepted historical teaching.

**FIGURE 4:**
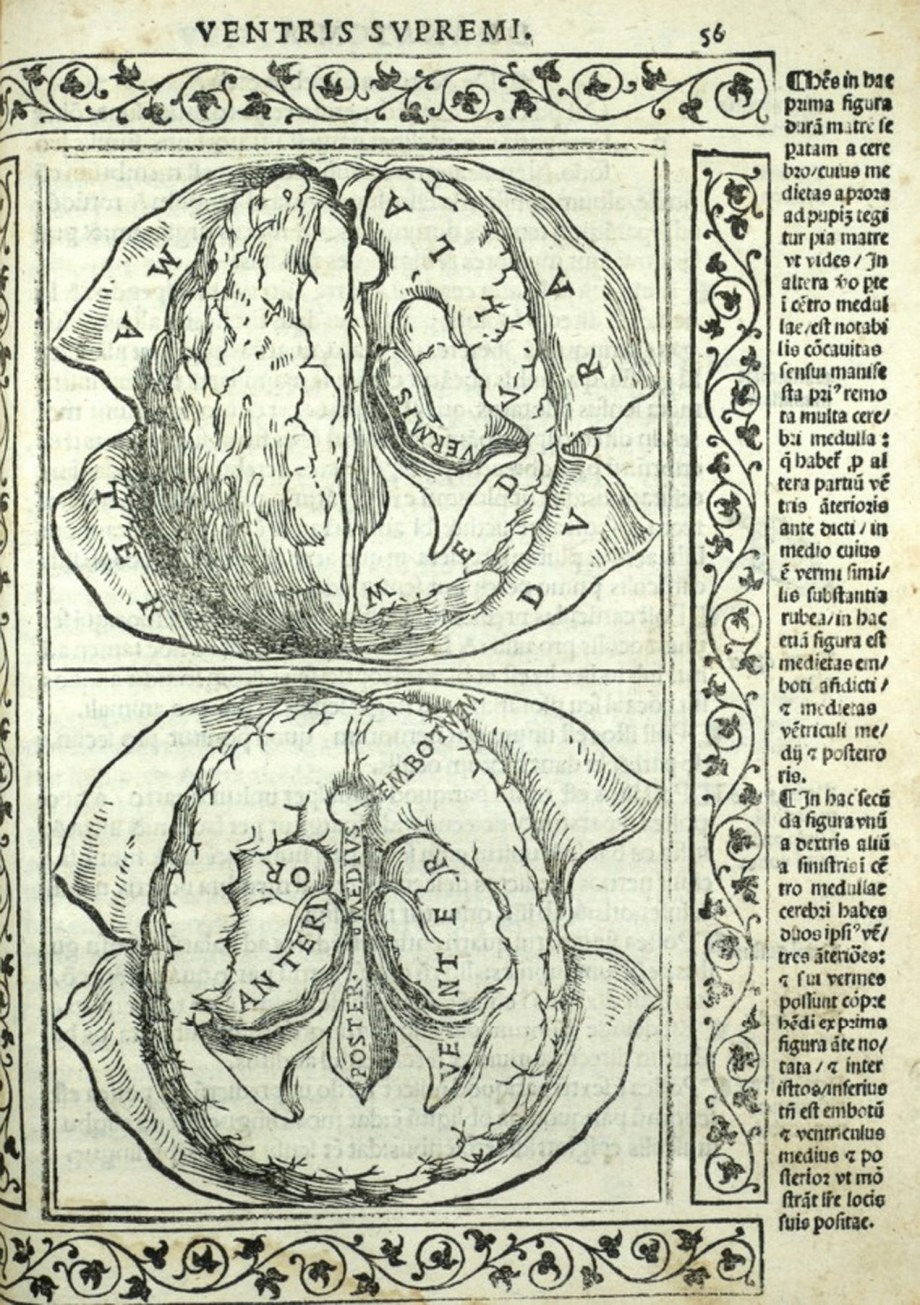
Carpi B (1523), *Isagogae Breves*, published Bologna, https://anatomia.library.utoronto.ca/islandora/object/anatomia%3ARBAI097 [accessed 6 august 2022].

It was Galen's belief that there was a ‘Rete Mirabile’ roughly translated to ‘marvellous network’ which transformed the ‘vital spirits’ (oxygenated blood) into ‘animal spirits’ which was a hypothesised agent that allowed nerve function (Quin, 1994). Berengario expressed doubt over the existence of the ‘Rete Mirabile’ (Parent, 2019), the existence of the structure in humans was concretely disproven by Vesalius (Figure 4).

### 3.4 Andreas Vesalius (1514–1564 CE) and De Humani corporis Fabrica

After obtaining his medical qualifications at the age of 23, Vesalius remained in Padua and lectured in surgery. This appointment came with the responsibility of delivering anatomical demonstrations. During this time, he dedicated himself to the study of human anatomy.

Vesalius' dedication to his trade is evident in his work. He wrote ‘He who espouses science must not marry a wife, as he cannot be true to both’ (Vesalius, 1543). In one case, ‘The handsome mistress of a certain monk of Sant'Antonio died suddenly and was snatched from her tomb by the Paduan students and carried off for public dissection. By their remarkable industry, they flayed the whole skin from the cadaver lest recognised by the monk who, with relatives of his mistress, complained to the municipal judge that the body had been [taken] from its tomb’ (Park, 1994). Surviving accounts from students of Vesalius paint a picture of an anatomist that taught the conventional curriculum but also challenged the status quo. Baldasar Heseler was a 32‐year‐old student when he attended an anatomy demonstration in Bologna in 1540, his notes of ‘a course of lectures on the Anatomy of Mondino di Luzzi’ demonstrated by Vesalius, who proceeded to correct the ‘many false opinions by dissection of three human bodies and six dogs… and showed us many things neither heard nor seen before’. (Poynter, 1964).

In 1538, he published the ‘Tabulae Anatomicae Sex’, a collection of six illustrated plates depicting subjects ranging from the human skeletal system to the major vasculature of the body. In the following year, Vesalius published the ‘Venesection Letter’, a contribution to an ongoing debate on the bloodletting procedure. Vesalius' letter highlighted his commitment to direct observation.

Although Vesalius had limited understanding of circulation as the Galenic teaching was very much still accepted. He went to great efforts to describe the exact arrangements of the azygous system of veins. This led to further study of the venous system by others, ultimately resulting in the observation of the venous valves, which would in turn formulate the foundations on which William Harvey postulated his theories of circulation. The ‘Venesection Letter’ is an important document, Vesalius questions whether ‘the method of anatomy could corroborate speculation’ (Saunders et al., 1982), cementing the importance of direct observation in anatomy. Within the ‘Venesection letters’, Vesalius writes ‘if the authority of Galen did not manifestly contradict me, and I am almost as afraid to argue about his authority as I would be tacitly to doubt the immortality of the soul in our holy religion’ (Vesalius et al., 1943 1539 [translated Saunders, 1943]). Following the success of the ‘Tabulae Anatomicae Sex’, Vesalius set to work on a greater work, titled ‘De Humani Corporis Fabrica’. An all‐encompassing textbook of human anatomy. The majority of the illustrations were made by Jan Stefan van Kalkar, an artist associated with the school of Titian in Venice.

Upon publication in 1543, ‘De Humani Corporis Fabrica’ revolutionised the way anatomy was taught forever. Previous publications on anatomy such as Mondino di Luzzi's *Anathomia* contained no illustrations. Some diagrams were added in retrospect by later anatomists such as Berengario da Carpi (Di Matteo et al., 2017). For the first time, the text describing the anatomical structures was placed next to large and clearly defined illustrations. This correlation of visual content with text reflects on Vesalius' methods of anatomical study, formulated on a basis of well‐documented direct and exact observations of the subject. The second edition of ‘De Humani Corporis Fabrica’ was published just over a decade after the first. Vesalius included further discussion of physiology, in particular descriptions of the venous valves. It is in this second edition that Vesalius distances his views from that of Galen. He confirms the absence of the pores, invisible to the naked eye, within the interventricular septum that Galen had hypothesised as the passage that linked the left and right sides of the heart.

There is no definitive answer as to how Vesalius' landmark publication was received. Some physicians were receptive to the work and its inquisition of accepted principles on anatomy. However, the editions of ‘De Humani Corporis Fabrica’ provoked a strong opposing response from some of the most renowned Galenist anatomists of western Europe. A growing body of scholars including Vesalius' former tutor Sylvius in Paris and his colleague Realdo Colombo from the University of Padua were extremely vocal in their criticism of Vesalius and his work. Sylvius published a pamphlet in 1551 where he spoke of his former pupil Vesalius as ‘a crazy fool who is poisoning the airs of Europe with his vapourings’ (Bakkum, 2011). Colombo who worked alongside Vesalius in Padua rightly criticised Vesalius on the elements of his work where there were mistakes. Vesalius defended these criticisms against him, falling out with Colombo, despite the Fabrica giving credit to Colombo for many of the discoveries contained within the publication.

Vesalius was invited to lecture at Bologna and Pisa, drawing large crowds, and defended his work (both rightly and wrongly in many cases).

**FIGURE 5:**
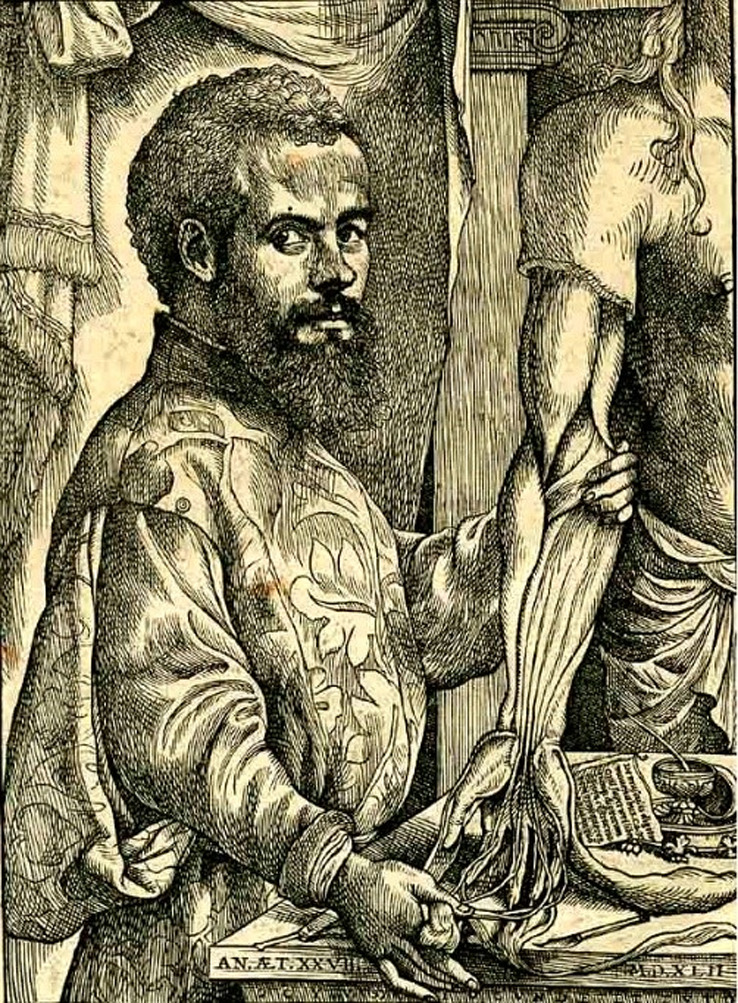
van Kalkar, J. (1534). *Portrait of Andreas Vesalius*. [Woodblock for printing] US National Library of Medicine.

Vesalius did not publish any other major works for the rest of his life. He entered service as the court physician of King Charles V of Spain. He held this position in the reign of Philip II. During this time, the work that Vesalius had conducted in Padua was evaluated by other physicians. Falloppio, who by now had taken up Vesalius' former position as the Professor of Anatomy in Padua, wrote a letter highlighting the mistakes of Vesalius and correcting some of his observations. When Falloppio died in 1562, Vesalius was on a pilgrimage to the Holy Land. He intended to return to Padua and resume as chair of anatomy at the university. On the way back from the Holy Land, Vesalius fell ill, and died at the age of 50, on the Island of Zante where he was buried in October 1564 (Figure 5).

### 3.5 How Vesalius and his contemporaries helped shape today's anatomy

Vesalius and his contemporaries were still trained in the medicine of Galen, and within ‘De Humani Corporis Fabrica’ are incorrect visuals and descriptions which allude to Vesalius' own lack of understanding of certain aspects of human anatomy and his reliance on Galen's teachings. Other physicians of the 16th century occasionally challenged the accepted understanding of human anatomy but arguably no significant progress was made during this time. However, their work paved the way for a new generation of anatomists and physicians that worked on the new principles and techniques laid down in the 16th century by figures such as Berengario da Carpi, Eustachi and Vesalius, to further the knowledge of anatomy and physiology with the scientific process. The ‘Fabrica’ of Vesalius may be seen as a pinnacle achievement of the era, the first major publication on anatomy since the time of Galen. For all its shortcomings, it was the defining anatomical text of the time. Revolutionary in its form, for the first time presenting clear illustrations and text descriptions side by side. The work represented new attitudes towards the study of anatomy, documenting what was observed, and learning by seeing and doing.

Vesalius was certainly not alone in this, his ‘Fabrica’ merely continued with the spirit of curiosity and discovery, in the direction set by Berengario, who in his published guide to Mondino di Luzzi's work, warned the reader not to be deceived ‘by some… who involve anatomy with authorities and not with observation’ (Montagu, 1955). Berengario also states in the same text that he would always accept the views of Galen except where observation is in discord with them, thus Vesalius was in no sense a revolutionary in his methods of working, Gabriele Fallopio in his ‘Observationes Anatomicae’ noted that ‘What (Berengario da) Carpi began, Vesalius perfected’ (Falloppio, 1561).

Key figures such as John Caius and William Harvey attended lectures in Padua, drawn by its reputation and revolutionary teaching. The groundwork laid by Vesalius and others spurred a new generation of scientific anatomists. Gradually, the observed anatomy was united with physiology. When William Harvey published his treatise ‘De Motu Cordis’ demonstrating blood in the animal body is in a state of ceaseless motion. His dismissal of Galen's theory of interventricular septum pores allowing blood to pass between was based on sound anatomical knowledge. Harvey understood the thickness of the interventricular septum compared to other tissue within the body and concluded ‘even if there were foramina or pores in this situation, how could one of the ventricles extract anything from the other… if both ventricles contract and dilate simultaneously’ (Hunter, 1931, p. 50).

### 3.6 Universities, their teaching and the establishment of anatomy theatres

The city states of northern Italy were an ideal breeding ground for radical reformation in higher education. These cities, largely immune to supreme imperial dominions or church governance allowed centres of academia to flourish. Copernicus studied medicine at the University of Padua and Galileo taught mathematics in Pisa. The cities of Bologna, Padua and Florence were some of the first documented to hold regular dissection demonstrations in a public university setting. Individuals working within these institutions are credited with many of the documentations that we use to form the basis of modern anatomical study. Living at the same time as Vesalius, Bartolomeo Eustachi (1520–1574) was a supporter of Galen's theories, he is credited with the discovery and accurate description of the connection between the nasopharynx and the middle ear which now bears his name. He produced illustrations on copper plates depicting anatomical structures. These illustrations were only published at the beginning of the 18th century, more than a hundred years after his death, his illustrations were less artistic than Vesalius' Fabrica but frequently more accurate in the portrayals of the human body (Adanır & Bahşi, 2019).

Another important figure living in the same period was Gabriele Falloppio (1523–1562) who held the position of Professor of Anatomy at the University of Pisa, Italy. As a physician, he was famed for his treatment of syphilis. Nowadays best remembered for his extensive documentation of the female reproductive system, he corrected Vesalius' misconceptions with regard to female reproductive anatomy.

Fabricius ab Acquapendente (1537–1619), a pupil of Vesalius, became an inspirational demonstrator of anatomy, his lectures drawing so many people that the University erected a wooden anatomy theatre for the raised capacity (Klestinec, 2004). This was the world's first permanent anatomy theatre built just over half a century after Vesalius performed dissections in a temporary structure. It was designed for public anatomical dissections and was constructed inside the Palazzo Bo, adjacent to the Hall of Medicine.

**FIGURE 6:**
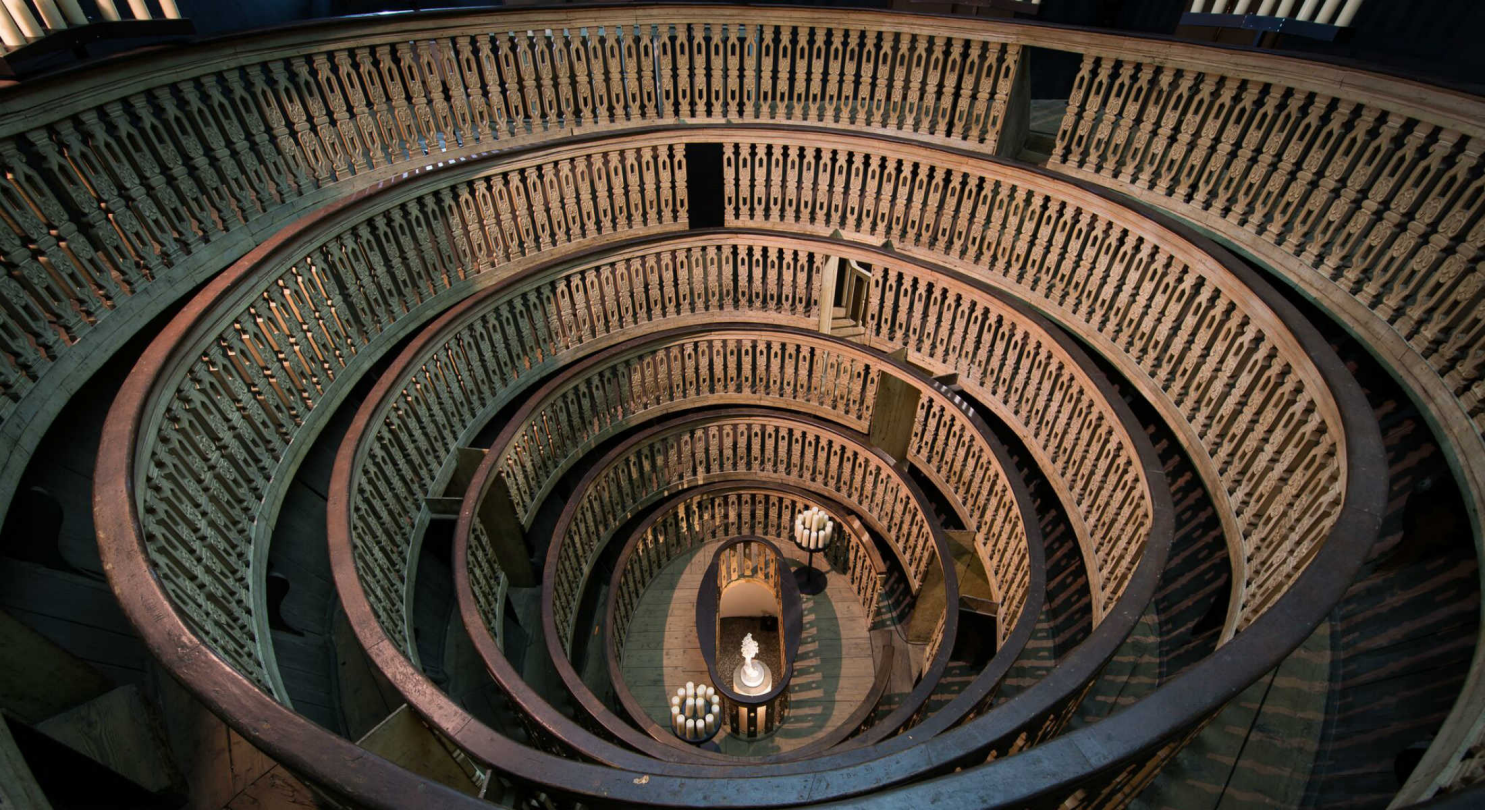
Hotson, H. the anatomy theatre at Padua, University of Oxford Library, https://www.cabinet.ox.ac.uk/anatomical‐theatres‐padua‐1594#/media=9660 [accessed 7 august 2022].

Initially, the dark‐walled concentric theatre had no windows, and the dissections took place under candlelight with each corpse taking several days to dissect. The practice of having purpose‐built anatomy theatres started in Padua, spreading to other cities and soon north of the Alps to other academic centres such as Paris and Leiden. At a similar time to Vesalius, an anatomy theatre was constructed in the Spanish city of Saragossa, with public demonstrations taking place under royal warrant (Martínez‐Vidal & Pardo‐Tomás, 2005). The construction of spaces specifically for the demonstration of anatomy reflects both the recognition of observation as a prime component of the scientific method as well as the changing attitudes towards human dissection (Figure 6).

### 3.7 Church, society and anatomy

The church and society itself played an important role in the progression of anatomical study during the times of Vesalius and his predecessors. Pope Alexander III in 1163 issued a statement ‘the church abhors blood’ (Somervillle, 2018) which led many to understand as forbidding the process of cadaveric dissection. Other papal decrees were misrepresented to suggest a ban on dissection in later years, likely affecting public attitude towards dissection of humans, although not halting dissection.

After Mondino di Luzzi's public dissection of a female criminal in the early 1300s, dissection and post‐mortem were a fairly common occurrence, though not widely performed in the public domain. Many accounts survive of wealthy families requesting post‐mortem examination by barber surgeons or physicians to discern the cause of death for their recently deceased relatives or forensic investigation (Park, 1994). Whilst the church was no concrete obstacle to the practice of human dissection, for many members of society, it held a suspicious and negative connotation. Many of the foremost anatomists were accused of scandalous and often irreprehensible acts. A rumour circulated that Fallopio had performed vivisection on twins both infected with syphilis (Shotwell, 2012). Similarly, Vesalius was subject to accusations that he had performed dissection on a Spanish aristocrat whose heart was still beating. Some accounts reason that Vesalius conducted his pilgrimage to the holy land as a form of penitence for this act. Whilst no proof exists of this event or the true reason for his pilgrimage to the holy land, it can be inferred that there was widespread distrust of anatomists and their practice. This likely would have stemmed from the fact that many dissections were performed in private and often in semi‐secrecy. As the steady demand for cadavers outgrew the quotas of executed criminals provided to anatomists at the time, other alternative ways of obtaining cadavers were taken (Ghosh, 2015). Although there was little resistance to the use of executed criminal corpses for dissection, on the occasions that students attempted to take corpses of those who died of natural cause or accidental death were faced with anger and disdain from the public as well as local church officials.

Dissection was often performed in the cold winter months, which would coincide with Carnival in late February (Ferrari, 1987). Often these public dissections would form part of the festivities of carnival, aiming to dispel the existing taboos. Over time, the public perception of anatomists evolved, especially as the practise of dissection became more widespread and integrated into the medical curriculum.

## 4 CONCLUSION

The advancements in anatomical knowledge cannot be attributed to a single person. It is a continuum of experimentation and discovery, which accelerated at the beginning of the 16th century. Each successive generation of anatomists benefited from the achievements of their predecessors. Vesalius' great work, ‘De Humani Corporis Fabrica’ was the culmination of not only his personal efforts but the innovations and changes in the practice of others working at the time. This provided successive anatomists, a strong foundation to dispel the incorrect theories of Galen which had been held as unquestionable truths for over a thousand years.

The work of Vesalius and his contemporaries provided the greatest leaps in not only understanding anatomy, but also the methodology in which to obtain it. These anatomists worked during a period of intense intellectual change in society, where once accepted facts were gradually proven or dispelled through methodical experimentation. Advancements in all areas of science, facilitated by changing societal and cultural norms allowed for the eventual union of observed anatomy and physiology to better our understanding of the human body and provide the basis for modern medicine.

## AUTHOR CONTRIBUTIONS

Author JX wrote the original essay which forms the basis of this article and the majority of the text, author SV contributed paragraphs relating to the development of higher education institutes' anatomical teaching as well as the development of the anatomy theatres. Both authors shared equal responsibility in editing and revising the article.

## Data Availability

No new data has been shared.

## References

[joa13773-bib-0001] Adanır, S. & Bahşi, İ. (2019) The giant anatomist, whose value is later understood: Bartolomeo Eustachi. Child's Nervous System, 37, 1–4. 10.1007/s00381-019-04107-1 30834964

[joa13773-bib-0002] Aird, W. (2011) Discovery of the cardiovascular system: from Galen to William Harvey. Journal of Thrombosis and Haemostasis, 9, 118–129. 21781247 10.1111/j.1538-7836.2011.04312.x

[joa13773-bib-0003] Ajita, R. (2015) Galen and his contributions to anatomy: a review. Journal of Evolution of Medical and Dental Sciences, 4(26), 4509–4516.

[joa13773-bib-0004] Bakkum, B. (2011) A historical lesson from Franciscus Sylvius and Jacobus Sylvius. Journal of Chiropractic Humanities, 18(1), 94–98. 22693484 10.1016/j.echu.2011.10.002 PMC3342831

[joa13773-bib-0006] Derenne, J. , Debru, A. , Grassino, A. & Whitelaw, W. (1995) History of diaphragm physiology: the achievements of Galen. European Respiratory Journal, 8(1), 154–160. 7744182 10.1183/09031936.95.08010154

[joa13773-bib-0007] Di Matteo, B. , Tarabella, V. , Filardo, G. , Mosca, M. , Lo Presti, M. , Viganò, A. et al. (2017) Art in science: Mondino de’ Liuzzi: the restorer of anatomy. Clinical Orthopaedics and Related Research®, 475(7), 1791–1795. 28054325 10.1007/s11999-016-5213-5 PMC5449319

[joa13773-bib-0008] Dunn, P. (2003) Andreas Vesalius (1514‐1564), Padua, and the fetal ‟shunts.”. Archives of Disease in Childhood ‐ Fetal and Neonatal Edition, 88(2), 157F–159F. 10.1136/fn.88.2.F157 PMC1721528 12598509

[joa13773-bib-0009] Falloppio, G. (1561) Observationes Anatomicae. Venettiis: Petrum Mannam, 1st edition. Padua, Republic of Venice: Marcus Antonius Ulmus.

[joa13773-bib-0010] Ferrari, G. (1987) Public anatomy lessons and the carnival: the anatomy theatre of Bologna. Past and Present, 117(1), 50–106. 10.1093/past/117.1.50 11617907

[joa13773-bib-0011] Frati, P. , Frati, A. , Salvati, M. , Marinozzi, S. , Frati, R. , Angeletti, L. et al. (2006) Neuroanatomy and cadaver dissection in Italy: history, medicolegal issues, and neurosurgical perspectives. Journal of Neurosurgery, 105(5), 789–796. 17121149 10.3171/jns.2006.105.5.789

[joa13773-bib-0015] Ghosh, S. (2015) Human cadaveric dissection: a historical account from ancient Greece to the modern era. Anatomy & Cell Biology, 48(3), 153. 26417475 10.5115/acb.2015.48.3.153 PMC4582158

[joa13773-bib-0016] Hunter, R. (1931) A short history of anatomy, 2nd edition. London: Staples Press Limited.

[joa13773-bib-0017] Kelly, K. (2014) Performing virginity and testing chastity in the middle ages. Abingdon: Routledge.

[joa13773-bib-0019] Klestinec, C. (2004) A history of anatomy theaters in sixteenth‐century Padua. Journal of the History of Medicine and Allied Sciences, 59(3), 375–412. 15270335 10.1093/jhmas/jrh089

[joa13773-bib-0020] Lloyd, G. (1973) Greek science after Aristotle. London: Chatto & Windus.

[joa13773-bib-0021] Martínez‐Vidal, À. & Pardo‐Tomás, J. (2005) Anatomical theatres and the teaching of anatomy in early modern Spain. Medical History, 49(3), 251–280. 16092787 10.1017/s0025727300008875 PMC1172289

[joa13773-bib-0022] Mavrodi, A. & Paraskevas, G. (2014) Mondino de Luzzi: a luminous figure in the darkness of the middle ages. Croatian Medical Journal, 55(1), 50–53. 24577827 10.3325/cmj.2014.55.50 PMC3944418

[joa13773-bib-0024] Montagu, M. (1955) Vesalius and the Galenists. The Scientific Monthly, 80(4), 230–239.

[joa13773-bib-0025] Parent, A. (2019) Berengario da carpi and the renaissance of brain anatomy. Frontiers in Neuroanatomy, 13(11), 1–14. 30814936 10.3389/fnana.2019.00011 PMC6381050

[joa13773-bib-0026] Park, K. (1994) The criminal and the saintly body: autopsy and dissection in renaissance Italy, 1st edition. Cambridge: Cambridge University Press, pp. 1–33. 11639270

[joa13773-bib-0027] Poynter, F. (1964) Andreas Vesalius of Brussels — 1514‐1564. Journal of the History of Medicine and Allied Sciences, XIX(4), 321–326. 10.1093/jhmas/xix.4.321 14215447

[joa13773-bib-0029] Quin, C. (1994) The soul and the pneuma in the function of the nervous system after Galen. Journal of the Royal Society of Medicine, 87, 393. 8046725 10.1177/014107689408700708 PMC1294649

[joa13773-bib-0030] Robinson, A. (2013) Galen: life lessons from gladiatorial contests. The Lancet, 382(9904), 1548.

[joa13773-bib-0031] Saunders, J. , O'Malley, C. & Vesalius, A. (1982) Illustrations from the works of Andreas Vesalius of Brussels. The anatomical drawings of Andreas Vesalius. New York, Bonanza Books: Distributed by Crown.

[joa13773-bib-0033] Shotwell, R.A. (2012) The revival of vivisection in the sixteenth century. Journal of the History of Biology, 46(2), 171–197. 10.1007/s10739-012-9326-8 22684269

[joa13773-bib-0034] Somervillle, R. (2018) Pope Alexander III and the Council of Tours (1163). Oakland, California: University of California Press.

[joa13773-bib-0037] Vesalius, A. (1543) On the fabric of the human body, 1st edition. Basel: University of Padua, Preface.

[joa13773-bib-0038] Vesalius, A. , Saunders, J. , O'Malley, C. & Clark, C. (1943) The bloodletting letter of 1539. An annotated translation and study of the evolution of Vesalius's scientific development by John B. deC. M. Saunders… (ed.) and Charles Donald O'Malley. From the divisions of anatomy and medical history. Oakland, California: University of California Medical School.

[joa13773-bib-0039] Von Staden, H. (1992) The discovery of the body: human dissection and its cultural contexts in ancient Greece. The Yale Journal of Biology and Medicine, 65, 223–241. 1285450 PMC2589595

[joa13773-bib-0041] Wills, A. (1999) Herophilus, Erasistratus, and the birth of neuroscience. The Lancet, 354(9191), 1719–1720. 10.1016/S0140-6736(99)02081-4 10568587

